# Can disability accommodation needs stored in electronic health records help providers prepare for patient visits? A qualitative study

**DOI:** 10.1186/s12913-020-05808-z

**Published:** 2020-10-16

**Authors:** Nancy R. Mudrick, Mary Lou Breslin, Kyrian A. Nielsen, LeeAnn C. Swager

**Affiliations:** 1 School of Social Work, Syracuse University, Syracuse, NY 13244 USA; 2 Disability Rights Education and Defense Fund, Berkeley, CA USA

**Keywords:** Electronic health record, Patient accommodation need, Primary care patients with disability

## Abstract

**Background:**

Embedding patient accommodation need in the electronic health record (EHR) has been proposed as one means to improve health care delivery to patients with disabilities. Accommodation need is not a standard field in commercial EHR software. However, some medical practices ask about accommodation need and store it in the EHR. Little is known about how the information is used, or barriers to its use. This exploratory-descriptive study examines whether and how information about patients’ disability-related accommodation needs stored in patient records is used in a primary health care center to plan for care.

**Methods:**

Four focus groups (*n* = 35) were conducted with staff of a Federally Qualified Health Center that asks four accommodation questions at intake for the EHR. Respondents were asked how they learned about patient accommodation need, whether and how they used the information in the EHR, barriers to its use, and recommendations for where accommodation information should reside. A brief semi-structured interview was conducted with patients who had indicated an accommodation need (*n* = 12) to learn their experience at their most recent appointment. The qualitative data were coded using structural coding and themes extracted.

**Results:**

Five themes were identified from the focus groups: (1) staff often do not know accommodation needs before the patient’s arrival; (2) electronic patient information systems offer helpful information, but their structure creates challenges and information gaps; (3) accommodations for a patient’s disability occur, but are developed at the time of visit; (4) provider knowledge of a regular patient is often the basis for accommodation preparation; and (5) staff recognize benefits to advance knowledge of accommodation needs and are supportive of methods to enable it. Most patients did not recall indicating accommodation need on the intake form. However, they expected to be accommodated based upon the medical practice’s knowledge of them.

**Conclusions:**

Patient accommodation information in the EHR can be useful for visit planning. However, the structure must enable transfer of information between scheduling and direct care and be updatable as needs change. Flexibility to record a variety of needs, visibility to differentiate accommodation need from other alerts, and staff education about needs were recommended.

## Background

A large and growing literature reports access problems people with disabilities experience in the course of receiving medical care, and the contribution of these access problems to health disparities and inadequate health care [1–3]. The problems include physically inaccessible medical buildings and physician offices, lack of accessible medical and diagnostic equipment, and inadequate procedures providers follow to ensure effective delivery of care [4–8]^.^ Where these types of barriers are present, patients will require modifications, called reasonable accommodations, in order to receive care. The specific accommodation that will enable a patient to access the health care facility and receive appropriate health services varies by the functional limitations of that patient. For example, a patient with a mobility impairment may need to be examined in a room with a height adjustable examination table, enabling the patient to transfer from wheelchair or scooter onto the table. A patient with an intellectual or speech impairment may require a longer than typical appointment slot to accommodate the slower pace of physician-patient communication. A Deaf patient will require the presence of a Sign Language interpreter. These kinds of modifications and preparations address the “accommodation needs” of persons with disabilities and enable their receipt of appropriate health care. Interviews with patients with physical, cognitive, intellectual, or psychiatric disabilities document that it is not uncommon to arrive for an appointment and discover that the medical provider is not prepared with the accommodations necessary to treat them [2, 5, 6, 9]. As a result, a patient may receive inferior care (for example, examined in a chair rather than on a table) or must make additional trips to the provider for the care that most people receive with a single visit.

Among the actions proposed to prevent these problems is the potential to embed patient accommodation needs in the electronic health record (EHR) so that providers can make advance preparation for the patient visit [10]. The wide implementation of electronic health records (EHR) following incentives in the 2009 Health Information Technology for Economic and Clinical Health Act (HITECH) and the Affordable Care Act would seem to offer the opportunity for patients and physicians to communicate and record needs useful to delivering care [11–14]. Although there are informal reports of medical practices that ask about accommodation needs and store this information in the patient record, there is little formal documentation regarding how accommodation information is collected and used, or the barriers or facilitators of its use.

There are two disconnected bodies of literature on the implementation of the electronic health record as discussed below. One set focuses on the broad implementation of EHRs and the barriers and opportunities for different population groups. However, these publications do not discuss the potential application for improving care delivery to patients with disabilities. The other literature consists of publications by the community of disability and health scholars who argue for the potential of the EHR, but whose publications do not include empirical examination of EHR implementation. To date, there is virtually no crossover between these literatures.

### Implementation of EHRs

The 2009 HITECH Act aims to promote the use of electronic health records in primary care practices through financial incentives for adoption, and later penalties for failure to engage in meaningful use of EHRs [15, 16]. The goal of the legislation is to improve health care quality, safety, and efficiency, and through meaningful use, improve care coordination, engage patients in their care, reduce health care disparities, and improve population health [15]. With this push, a number of topics have been the focus of a burgeoning literature. Numerous publications speak to the advantages of implementing an EHR with the possibility of reduced error, greater efficiency, more seamless communication and coordination across multiple providers serving the same patient, easier monitoring of patient outcome measures over time, and greater transparency with patients who can access their own test results and other records through a patient portal [17, 18]. Other publications consider the challenges to developing and implementing EHRs, including decisions about what data items should be included, how much detail, how to incorporate nuance and narrative information, how to assure needed flexibility, and how to overcome doctor and patient resistance [19, 20]. Publications also consider doctors’ concerns that the computer screen and keyboard will diminish patient-doctor communication, or reduce patient satisfaction [21, 22]. While some of these publications consider which population characteristics should be included in the EHR structure, for example, age, race, ethnicity, spoken language, and marital status, disability status or accommodation needs as potential data fields are rarely mentioned [17, 23, 24]. Recent additions to the health information technology literature have discussed how population information can be produced that could be used to identify and address health disparities [25, 26]. However, people with disabilities are not identified as a health disparities group in these articles.

### Potential of EHR-embedded disability information

Researchers concerned with the barriers to health care experienced by people with disabilities describe potential benefits of EHRs, such as information about accommodation needs during a medical exam [11, 12, 14]. These needs could include an ASL interpreter, a longer appointment time, assistance with mobility, or scheduling for the one exam room with the height adjustable exam table. Among potential problems are concerns that the patient portal software will not be easy to use and accessible, especially for patients with visual or intellectual impairments, and that the EHR architecture might not be able to capture the complexities and interacting factors of health care problems [13, 27]. Few studies have actually observed the daily workflow of a medical practice to understand the use of the EHR with patients in general, or with patients with disabilities [13, 18, 19, 24, 28].

The aim of this exploratory research is to learn whether and how information about patients’ disability-related accommodation needs, stored in electronic patient records, is used in a primary health care setting to plan for care. Our specific foci for health center staff were: (1) do they use the accommodation need information in the EHR to assist visit planning; (2) what are barriers to using accommodation information in an EHR; and (3) and what information, format, and timing of information would be of most help in preparing to treat patients with accommodation requirements. As part of exploring the collection and use of accommodation need information, a fourth focus sought to know (4) if patients had expectations of disability-related accommodations because they indicated a need on the registration form.

## Methods

### Study design

This exploratory-descriptive study is grounded in the framework of the Normalization Process model, an implementation model for factors affecting the routinization of new practices, actions, or ways of organizing work in healthcare settings [29]. Its cognitive participation and collective action constructs are especially applicable to our problem and setting because we are exploring the utilization of a process innovation. Qualitative methods are used to collected data from the staff and patients of four clinic sites of a single health care organization. Data were first collected from clinic staff with focus groups, followed by a small number of brief phone interviews with purposefully selected patients. Institutional Review Board (IRB) approval was obtained for both data collection efforts before any data were collected. An exploratory design was chosen because there was no prior empirical research based upon observing the intersection of patient accommodation needs with primary care electronic records. Consideration of patient accommodation needs was not restricted to specific disabilities or kinds of impairment to enable the identification of unexpected areas of need and information use.

### Study setting

The setting for this study is a Federally Qualified Health Center (FQHC) located in Northern California. Federally Qualified Health Centers offer community-based primary care to underserved areas with a sliding fee scale for low income persons in return for special financial support from the U.S. government [30]. Across the U.S. there are 1362 FQHCs delivering care in 11,744 sites. In 2018 FQHCs delivered care to 28 million persons, 68% of them below the U.S. poverty threshold, 63% from a racial or ethnic minority group, 48% enrolled in Medicaid (the government health insurance for low income persons), and 23% uninsured [31]. While there are no national data regarding the percent of FQHC patients who have disabilities, the documented association of poverty and disability suggests that many FQHC patients may be persons with disabilities [32].

Starting in 2014, nine clinics of the Health Center studied here added four functional impairment questions to the patient management record that it stores electronically. The intent was to be able to alert primary care staff about a patient’s accommodation need with the goal of facilitating quality care. Patients were asked to indicate at initial intake whether they needed (1) a Sign Language interpreter (asked as part of language interpretation need), (2) assistance with mobility, (3) support for low vision or blindness, or (4) a long appointment. In 2015, approximately 9% of patient records indicated an accommodation need [14], although the Health Center did not track whether staff were noting and using the information to plan for care. In 2016, aware that the Northern California Health Center was one of the few health systems that had integrated accommodation need fields into their EHR, the researchers approached the Health Center and were given permission to conduct an exploratory study of staff use of the information and any barriers or suggestions for improvement.

### Focus group methods

#### Study participants

To learn about the use of the accommodation information, four focus groups were conducted with staff from four Health Center clinic locations. The staff were invited to voluntarily participate in the 90-min focus group session via printed announcements posted at the clinic sites and put in staff mailboxes and in-person discussion with staff. The flyers stated the topic was “Understanding how disability-related accommodation needs are identified and provided in health care.” Interested persons were instructed to telephone or email one of the researchers. Everyone who responded was included in a focus group. Altogether, there were 35 participants in four focus groups (with 10, 4, 9, and 12 participants).

#### Data collection

Focus group sessions were held at two clinic sites at lunchtime or at the end of a workday, with refreshments. Each participant gave oral consent to participate before the start of the session and received a $100 honorarium. The same individual facilitated all four groups. The focus group interview guide asked participants to discuss: 1) how they learn about patient accommodation needs and how they use their electronic patient records, including the four accommodation need questions, 2) how they plan for the day’s appointments and the chief logistics challenges and issues with patient flow, 3) what they would like to know about patient accommodation needs, and when, and 4) where they think such information should be located for best use. Two members of the research team developed the interview guide for this study. Some questions were worded closely to the study aims and some questions asked how a potential situation would be handled (the focus group interview guide is available as a Supplementary document). Each session was audio-recorded and then transcribed. No information about the participating staff members, such as education, years of experience, age, or race, was collected. The only identifying information collected was work role (e.g., nurse, scheduler, etc.).

#### Data analysis

Transcripts from the focus groups were uploaded into the qualitative analysis software, NVivo, to enable connection of text to a code and later grouping and synthesis. Structural coding, considered especially appropriate for multiple participant transcripts from semi-structured interviews, was the primary coding method [33]. Structural coding involves developing a code-phrase that reflects content or a concept expressed in response to a specific research question or topic of discussion. To enhance the neutrality of the coding, two persons who were not present at the focus groups did the initial coding. Each transcript was coded by the first individual who developed a set of code phrases (code categories) to capture different elements of the content of statements. The second coder independently coded the transcripts using the set of developed codes. Comparison between the two sets of coded transcripts after the first pass found a 95% or higher degree of agreement for the assignment of text phrases to each of the major code categories. The second coder then examined the areas of disagreement and the definitions of the codes and adjusted some codes and left other points of disagreement unchanged. A group consisting of the two coders, the focus group facilitator, and another member of the research team, then reviewed the codes and their linked text and grouped the code categories into the set of key themes. The involvement of the focus group facilitator and the fourth researcher at this point gave confidence to the reliability of the link from transcripts to themes. From the themes, the group reached consensus on the main findings.

### Patient interview methods

#### Study participants

Interviews with a purposefully selected set of patients were conducted following the completion of all focus groups. The target was to interview 12–15 persons. Ninety-seven adult patients were selected based upon a set of criteria and mailed invitations to participate in a short phone interview. The primary criteria were that the patient (1) had an accommodation need listed in their electronic record and (2) had visited the Health Center for care within the past 6 months. With the researchers’ instructions, a Health Center administrator identified the records of eligible patients, also selecting to ensure that the set of patients invited was diverse by race/ethnicity, age, sex, and type of impairment. Patients were mailed an invitation to participate in a phone survey using the researchers’ stationery and envelopes. To keep patient identities confidential, the Health Center put the address labels on the researchers’ pre-paid envelopes and mailed the invitations. Persons willing to be interviewed were instructed to contact the researchers directly so that the Health Center never learned who responded to the invitation. Thirteen persons agreed to the 30-min interview. After starting the interview, it became evident that one of the respondents did not have any disabilities, perhaps having marked the form in error. Thus, the final number of patient respondents is twelve (12% response rate to the interview invitation).

#### Data collection

Interviewees and the researchers agreed on a day and time for the interview. Before the interview, patient-respondents who had access to email received the consent form electronically to sign and email back. If they did not have email, consent was obtained orally at the start of the interview. After the interview each respondent received a $50 gift card, sent by US postal mail or email. One interviewer audio recorded interviews, and these were later transcribed. The other interviewer created the transcript by typing answers verbatim during the interview. Interview methods were modified as needed to accommodate patients’ disabilities. For example, one respondent, who knew her speech was difficult for others to understand, had an assistant familiar with her speech patterns on the telephone who restated her replies when needed for clarity. The mother of a patient with an intellectual disability, who could not speak for himself, was the respondent in another interview. A third respondent requested that the interview occur through email exchanges for reasons related to her disability.

The patient interview guide developed in iteration by two of the researchers had 8 closed-ended and 2 open-ended questions, with follow-up probes possible for any of the questions. Respondents were asked how long they had been patients of the clinic, and whether their most recent visit was for a routine check-up or follow-up for a continuing condition. Patients were asked about their functional limitations based upon the six limitations that comprise the American Community Survey disability questions [34].^1^ They also were asked what accommodations they required when receiving medical care, and whether they recalled indicating their accommodation need when they signed up for care. Finally, they were asked how important was special assistance or arrangements to their sense of receiving good medical care.

#### Data analysis

Transcripts were created from the audio-recorded and typed interviews. The closed-ended questions were tallied, and two persons independently hand-coded the open-ended questions. Because interviews were intentionally brief, many of the open-ended questions produced short answers. Thus, the interviews did not generate a lot of qualitative data. Structural coding was utilized to create code-phrases for the content of the responses. One coder identified key phrases from the responses to each open-ended question and from this developed 10 code categories applied across all the questions. A second coder independently identified key phrases from the responses and assigned them to these code categories. Disagreements were resolved in conversation with the full research team, and several categories were combined, and one category added. The research team extracted key themes from the codes and developed consensus on the main findings.

## Results

### Focus group interviews

Thirty-five staff members participated in the focus groups. Groups were a mix of staff that included a doctor, nurses, medical assistants, social workers, referral specialists, psychologists, triage assistants, managers, administrative assistants, and receptionists. (see Table 1). We do not know how many staff members saw the focus group recruitment materials, but the potential could be 278 persons (producing a rough response rate of 13%). Staff members described their work roles as belonging to the “front office” or the “back office.” Front office staff are the non-medical personnel who schedule appointments, greet patients at reception, make calls for referral to other providers, do community outreach or are part of management. The back office staff are the clinical staff who treat patients, and this includes doctors, nurses, medical assistants, psychologists, and social workers. Both front and back office staff have a role in planning and delivering patient care. All of them worked with parts of patient electronic records.

**Table 1:** Health Center Roles of Focus Group Participants

Role Title	Number
Front Office Receptionist	4
Triage and Referrals Specialists	5
Registered nurse	3
Medical assistant	10
Physician	1
Social worker/psychologist	3
Community health worker or fellow	4
Manager	3
Other	2
**Total Focus Group Participants**	**35**

Patients can initially register with the Health Center or make appointments through several avenues. One option is a central registration and scheduling phone number that serves all clinic sites. Another option is for a patient to call a particular clinic site and speak with someone in reception. Persons who just show up at a clinic (walk-ins) can be registered or scheduled on-site. The four questions about functional limitations that tap at accommodation needs are part of the information gathered at initial registration.

Five major themes emerged from analysis of the focus group interviews. These themes provide insight about when and how providers learn of their patients’ accommodation needs, how different professionals within a health setting use electronic patient records, and improvements in the communication structures around accommodations that might enable them to be more prepared. These themes are listed in Table 2 and each is elaborated in the discussion that follows.

**Table 2:** Key Themes from Health Center Focus Groups

Key Themes	
1: Accommodation needs generally are not known in advance of the patient’s visit.	
2: EHR systems offer helpful information, but with usage challenges and information gaps.	
3: Accommodations do occur at health visits but often are developed at the time of the visit.	
4: Knowledge of a regular patient is often the basis for advanced accommodation preparation.	
5: Providers acknowledge the benefit of preparation for accommodation needs and are supportive of methods to enable it.	

#### Theme 1: accommodation needs generally are not known in advance of the patient’s visit

Most of the time, staff do not know accommodation needs before the patient’s arrival, except for the need for American Sign Language interpretation which is arranged by the front office. Most of the clinical staff responded that they did not know ahead of time. Some offered that patient charts sometimes contain a note about accommodation but stated that they generally review the chart the morning of the appointment or just before the patient arrives.

*Doctor: No. I’d say 90% of the time, no. Sometimes we do chart preps, so we get a schedule, and the patient has a couple lines of what they’re coming in for. And so sometimes that will say whether they need an interpreter, depending on who took the appointment, … Or if you look in the patient’s chart ahead of time because you know they’re coming, then you might find out something based on that. But for the most part, I don’t look at those schedules until maybe the day before or the morning of the appointment.*

Scheduling staff indicated a greater awareness and advanced knowledge of patient needs, particularly if the person needed ASL interpretation. For them, accommodation need might appear as an alert in the scheduling software. These staff also noted that the needs of a patient seen regularly could be planned for because of knowledge of the patient.

*Receptionist: If they registered through the call center and if they need an interpreter they email me and let me know that they need an interpreter and then in that I request an interpreter for that particular date but I have to have 48 h’ notice in order to get an interpreter for the patient … And if they need assistance as well too, the call center, they’re supposed to let us know, … that this patient needs assistance with coming up. They can’t make it up the stairs. They can’t do the elevator. They need some type of assistance and then again, we’ll let them know, we’ll let the MA float know what’s going on.*

The need for language interpretation seemed to be the primary information element recorded in the electronic scheduling record that is noticed and acted upon. In discussing this, staff often did not differentiate between interpretation for ASL or for persons who do not speak English. The second most frequent alert element mentioned by the clinic staff was a patient’s use of a wheelchair. While it does not appear to trigger planning in advance of the medical visit, it may be noticed in the morning schedule review. In this way, awareness can affect the organization of that day’s patient flow for the larger exam room or additional staff availability to assist when the patient requiring these accommodations is ready to be seen.

*Receptionist: If the patient calls you on the phone and they’re using an interpreter, sometimes the call center will put the alert and notify us. Other times, most of the times they don’t do it until the day before the appointment when we call to confirm, that’s when we have to find out that they’re, you know they’re ASL, or they need an interpreter, or they’re coming in a gurney, or they have a case manager. And that causes problems because we can’t plan ahead to be able to get the right person to assist in the appointment if they don’t bring their own person.*

*Center manager: I’ve seen other alerts but usually it’s for language interpretation or if there’s a wheelchair needed...It does say if they have a service dog, there’s an alert for that. ... I think most of the clinics once we see the patient we tend to add more alerts to things and I would think that you would because you deal with a very particular set of patients.*

#### Theme 2: EHR systems offer helpful information, but with usage challenges and information gaps

The electronic patient information systems offer helpful information, but their structure also creates challenges and information gaps regarding patient accommodation needs. The scheduling staff and the clinical staff described the ways in which they used the electronic information about a patient to prepare for delivering care. The scheduling staff regularly use information indicating the need for ASL interpretation as a flag to make the arrangement. Where there were notations about needing a longer appointment, they did their best to schedule that. One strategy they shared was to schedule a patient needing more time before the lunch break, so some extra time could be available. The medical assistants and the doctors described looking over the schedule of patients the day before or morning of the patient appointments as part of chart preparation. The medical assistants used the information noted about patient accommodation needs to plan for who needed which exam room and when extra assistance might be needed.

*Medical assistant: Sometimes it’s easy like if the alert is there. The order may notify and also because we also check in all the alerts. We’re trying to just plan for a patient in a wheelchair and if coming for a pap we know what the patient is coming so it’s easy to see the alert that’s going to require a long appointment.*

Many primary care organizations started using electronic records for patient registration, scheduling, and billing before the push to make the complete medical record electronic. The electronic medical record was later added on as a related, but somewhat separate system. As a result, the scheduling staff in many primary care settings, including this site, work with the “patient management record” (EPM) and the clinical staff work with the “patient medical record” (EHR). What became clear in the focus group discussion was that information obtained and stored in the management record, for example the need for mobility assistance, might not be transferred to patient medical record portion. And while staff have access to both the EPM and the EHR, there is a division by function such that scheduling and reception staff mostly use the EPM component, while the clinical staff mostly stay within the EHR component. Thus, an accommodation need observed during the medical exam might not get recorded into the EPM so it is seen when the next appointment is scheduled. This issue became clear in the response to the question of where accommodation information is recorded. The registered nurse is referring to the EPM.

*Registered nurse: You can put anything in there. It’s free text. It could say, patient needs to be roomed with a lift. You could write anything. It’s not just like a limited selection of text in there. But that’s only scheduling. And providers don’t do that. There’s only a certain subset of like the MAs* [medical assistants] *do it. And it’s not consistent that it’s been put in there either necessarily.*

*Interviewer: And will the treating physician see that information?*

*Registered nurse: No. That’s the problem. It’s the staff who’s scheduling. Only the people who schedule. And they don’t schedule for themselves.*

*Doctor: So sometimes if we know the patients, like [Registered nurse] said, if we’re scheduling them for follow-up appointments and something hasn’t been done for them, I’ll ask our MA to say, can you put in the system that they need this for the next appointment so they can do that? But I don’t know how to do that personally. And then we don’t see those alerts because we don’t do scheduling. So it’s a separate system.*

A medical assistant offered another example of inefficiency from miscommunication tied to the two systems. In this case, the clinical staff member has to re-do the schedule for the appointment because of the patient’s other regularly scheduled care. From the patient’s perspective, it probably seemed surprising that the medical provider was not aware of which days were dialysis days.

*Medical assistant: See, front desk sees that but on the MA end, we don’t see that. I don’t see oh this patient can only come in Monday, Wednesday they have dialysis Tuesday, Thursday. So, if I make an appointment for that patient on a Wednesday, they’re like, no I can’t come that’s dialysis day. And then I have to go back to my desk and re-give appointment and they’re like I’ve been telling you guys this. And we’re like, well sorry because we don’t see that on our end, only the front desk sees that. Especially if they see a different provider, it’s not their regular provider.*

The need for an ASL interpreter, or other accommodation, can be inserted in the electronic record so that it pops up as an “alert” to the user. Most of the alerts about accommodation pop up when scheduling. However, these accommodation alerts may not pop up in the medical record (EHR).

*Receptionist: Our only problem with the alerts is that we have two systems in the front, we use one in the back office for the medical assistants, they use another one most of the time. So, if we put an alert in the front part of an EPM, when you go to EHR and the person only uses EHR all day, they don’t see the alerts. They don’t know that the patient needs a Sign Language interpreter or has mobility issues.*

*Interviewer: Okay, who sees EHRs and who sees EPMs?*

*Receptionist: We all see it, but if you’re working on it like for a medical assistant, the only time they see the alert is if they go back to make an appointment. So, at the end of the appointment. They won’t see it at the beginning of the appointment when they put the vitals in and they have the patient in the room.*

The manager of a different clinic site provides a similar description of the operation of the two parts of the patient electronic database.

*Center manager: There’s a very particular set of people that deal more, mostly with the EPM, the practice management part* versus *the health records. But everybody has access to both so the goal, it would be fantastic if everybody kind of just looked at both. Because I notice that there are times that there are alerts in EPM that don’t make it over to EHR so then the providers aren’t aware that there’s a particular concern about, some, a patient or a need … We tend to focus on one particular, one of the two.*

Staff offered additional observations of how the complexity of the EPM and EHR system makes it difficult to obtain the information related to patient accommodation needs. One issue was the large number of different alerts that pop up, intended to make the provider aware of important issues for that patient. However, some felt that there were so many alerts that their impact was muted. Others noted that the accommodation information in the alert was too brief to usefully assist preparation. The criticism of too much brevity also included the four accommodation questions completed at registration.

*Triage assistant: So essentially, it’s part of when they’re going through the registration form, it’s a part of the questions. They’ll say, do you need assistance during appointments? And then those four things come up, which they can click. But you can’t specify, but you can only click low vision blindness, language, mobility assistance.*

The health provider is large enough to have several persons whose job titles are “referral specialist.” These individuals receive notification from the doctors to set up a referral with a medical specialist. In the focus groups, referral specialists indicated that information about accommodation needs does not accompany a referral request and they often do not see it in the parts of the database they use. The referral specialists indicated that they have another electronic system for referrals, and it does not contain the accommodation alerts activated in the EPM/EHR system. Referral staff said they could use the EPM and EHR to read about the patient and identify accommodation needs, but that the volume of referrals they are sent, and the speed with which they feel they need to arrange them, discouraged them from taking the time to dig deeply. The referral specialists agreed that at times this created problems, acknowledging instances in which they had made a referral appointment only to hear from the medical specialist provider or the patient that the setting was inaccessible or that that the provider was unable to accommodate that patient and provide care [35].^2^

*Referral specialist: I do referrals and I don’t look at alerts because well I am with EHR and I use [product name] and so I don’t know when a patient needs special accommodation. I have no idea unless I go through the chart and read and then I’ll find out. I refer patients out to see specialists and some of the time the places that I refer them to, they don’t offer special accommodation so I’ll get lots of the patients calling back or the place calling back saying we don’t have wheelchair access. We can’t see your patient. I just had a patient recently that is blind and deaf. I did not know that until I got a phone call from the mother of the patient and that’s when I had to do all the specific arrangements for the patient. So I’m just wondering if there’s a way other than the alert to know ahead of time that the patient requires these accommodations.*

#### Theme 3: accommodations do occur at health visits but are often developed at the time of the visit

Accommodations for a patient’s disability are made during health visits, but many of them are put in place at the time of the medical visit as a need is recognized. Even if they had not been able to make advanced arrangements for a patient’s accommodation needs, the staff members in the focus groups described how they worked to put a needed accommodation in place “on the fly.” Many of the situations they described involved someone with a mobility impairment. The receptionists described alerting the medical assistants when it was clear an extra person would be needed to assist a patient with transfer. A medical assistant described going out to the street when the patient called needing help getting out of the car and into the office. In the case where the ASL interpreter did not show up for a Deaf patient, rather than make the patient reschedule, they described efforts to communicate with writing or by using a staff member who had some knowledge of ASL. Focus group members acknowledged that the time required to implement these “on the fly” accommodations sometimes affected the planned schedule, but they did not view this as a big problem.

*Administrative assistant: It might throw off a little bit of the flow, but then again you have the no-shows or the cancellations, so it balances. Or we make it work.*

One person described the schedule as “an outline to the essay” and the others in the group agreed. Some of the case examples they offered indicated dedication and creativity.

*Receptionist: We have a couple of patients that come to us from care homes so sometimes we’re not aware when they’re coming … and there are times when we are not prepared for them because when they’re coming in, they’re coming on their gurney so we have to rearrange the area to make sure that they’re able to come in and go to the room where they have them, where their appointment is taking place... I think if we had a little bit more heads-up that that person is coming then we can be prepared and not have, a transportation or the paramedics waiting out in the hallway, for us to kind of move things around.*

Beyond preparation for patients who required ASL interpretation, it seemed there was little advanced preparation of communication methods or materials that might be useful to patients with visual or intellectual disabilities. Staff members reported working out alternative formats for communication as they are recognized, and offered a number of examples of the workarounds they use to meet this need.

*Staff psychologist: Because people may not know how to read, or they’re fluent in English but they’re not able to read English, YouTube is your friend. Everything under the sun is on YouTube … So if there’s health information that someone needs and printed and the reading is an issue whether it be the low-vision or the inability to read, I press “go” you know, and I have YouTube things going, … If there’s any questions about someone’s cognitive abilities or abilities to read or abilities to take in information in a print format, or [inaudible], I YouTube it, immediately. And that’s the saving grace in the worst times.*

At the same time, the medical providers understood that a makeshift arrangement may not be ideal from a quality or patient-centered perspective.

*Registered nurse: Yeah, that would be a work around. I mean, that’s my stock and trade as a nurse, how to educate patients in a way that will make sense to them, whether it’s finding a YouTube video [inaudible] that shows them how to use their inhaler. Or whatever might be. In that case, you know, I would ask the patient if they have a recording capacity on their phone and read it and record it for them. Or if they have an answering machine at home and I can call them, read the instructions to them, or, you know, but it would be a MacGyver.*

Responding to an unexpected accommodation need also affects the care experience of other patients. It can result in a longer wait to be seen or other modifications.

*Medical assistant: But just say for instance, there’s two that come in with the wheelchair, and like [Receptionist] said, if we’re already using two of those rooms for patients who are nonwheelchair patients because they’re regular rooms, we have to switch patients, ask patients to get up, move to another side of the hall, … yeah musical chairs with them. Just to make sure everybody is being seen.*

#### Theme 4: knowledge of a regular patient is often the basis for advanced accommodation preparation

Provider knowledge of a regular patient is an important source of information for making advanced accommodation preparation. Although the schedulers, receptionists, and the clinical staff all spoke about their use of the accommodation need information in the electronic patient record, the discussion indicated that they rely a great deal on what they know about the patient from prior medical visits.

*Eligibility specialist: When you build a relationship with your patient, you just automatically know. Like this is what we do when we’re done with them, you know, or schedule the interpreter or whatever is needed. Or if they need a longer time. That’s just what we do automatically. Because most of our patients are like ongoing patients that come like twice, three times a week. So we just automatically know, we know them by name. It just comes naturally.*

*Medical assistant: Like [inaudible] patients sometimes they take longer to speak. Yeah, one of the nurses, she has like patient cards, specifically for a provider because she knows the patient that we work with. But patient cards for the patients who like point out all different medications, especially if she knows what medications they are on to help with the [inaudible]. ..But if it’s someone who is slow speaking then we just take our time with them.*

*Triage assistant: I think it’s easier like once we know the patients, and like, oh, Mr. Such and such is scheduled today. So we can be sure when we see that person rescheduled. But for new patients, which differ, and we’re still learning about them and they’re learning about us.*

The doctors, nurses, and clinic receptionists talked about patients whose care they knew usually requires more than the standard 15-min visit. If they were involved in setting up the next appointment, they would strive to place it where there was the possibility to run beyond 15 min. This knowledge was not necessarily recorded electronically. Thus, if the patient used the call center to schedule an appointment, the time offered might not allow for flex, and an additional appointment could be required. Knowledge of a patient can play a role in accommodations that are more idiosyncratic, such as ensuring the availability of the nurse who is able to help a patient with behavioral health limitations to remain calm.

Knowing the patient also meant knowing about variations in the standard accommodations. One staff member gave an example of parents who insist that they transfer their daughter to the exam table, rather than allow the center staff to do it. In another example, a medical assistant described the downside of variation where a Deaf patient prefers using a family member to an ASL interpreter arranged by the Health Center.

*Medical assistant: And sometimes with the patients, some patients don’t disclose their disabilities, so we wouldn’t know until we see it. This brings up one of my patients - they have a family member that comes in and does ASL for them, we didn’t know that at first and they don’t use an interpreter, when we got to give them one, so they want to use the family member. Which takes time because the family member doesn’t know all the words.*

The more often a patient was seen by the health center staff, the more that needed accommodations were known and worked into visit planning by providers when they reviewed the patient list for that day or the next. The call center schedulers and referral specialists have responsibilities for assuring accommodation is present, but they have fewer opportunities to know a patient beyond information present in the electronic record.

#### Theme 5: providers acknowledge the benefit of preparation for accommodation needs and are supportive of methods to enable it

Provider staff recognized the benefits to having accommodation information in advance and were interested in a systematic protocol to make this occur. Health Center staff were in agreement that having information about accommodation needs in the electronic patient record is helpful. Many stated that the four questions currently in use should be kept. However, there were a number of suggestions for additional information and for ways to ensure the information was brought to the attention of everyone.

*Medical assistant: I think like the pop up questionnaire, just straightforward, like a four-question questionnaire would be helpful that way we can all see it from all in both the front desk, MA and the provider perspective. ... Like we’re trying to improve the clinic so much as like the whole like you know be fancy get new chairs and all this, and TVs and all this. But I feel like you still have to accommodate our patients and if we’re not doing that then we’re not going to be successful as a clinic. You know and as an MA, I can’t fully do my job if I don’t know. I feel bad if I don’t know, this is my patient coming in with a disability and they’re looking at me like you’re supposed to know that.*

*Registered nurse: … frankly, the [EHR] chart that we use currently, which is [product name], I go into the demographics all the time hoping that there’s going to be something helpful there, and I’m often disappointed by what I find, either because people don’t put in--it’s very clumsy to put in information. And then it doesn’t always reveal in a very easy way on that banner. I mean, I was kind of thinking ideally what I would like was that when you open up a patient, there’s some sort of overlay of what the patient desires and what they want you to know about them and keep in mind for their visit before you get into the details. … We are the ones interpreting what they say or putting it in into our own language. And I would really like something more in the patient’s voice around things that they need in terms of accommodation instead of this generic drop-down menu choice.*

There was variation across the staff members regarding what level of detail about patients’ accommodation needs was necessary, especially given how much other information needed to be entered in the record. There also was discussion regarding the format that would best bring that information to everyone’s attention. The system already pops up a number of other alerts, and so there was concern that one more alert could be missed or ignored. One staff member mentioned an electronic record system she had seen at a conference that used color coding to attract the user’s attention to the different types of information. Others in the group expressed interest in this idea.

Finally, while the focus group interviews were not intended to sensitize staff to disability and accommodation, in the course of the group discussions, participants commented on what they were becoming aware of from the discussion. Persons with management responsibilities noted things they could implement to improve access. Some staff members expressed their need for, and interest in, knowledge for “disability cultural competence.” Others spoke about the need for more accessible equipment at their site. There were discussions in each of the focus groups about the need for more long appointment slots. In fact, there seemed to be general confusion regarding whether any long appointment slots existed, whether staff were “allowed” to book two 15-min slots to create a single 30-min slot, and how such additional time should be coded for billing purposes.

### Patient interviews

The 12 patients interviewed were persons with moderate to severe functional limitations. Table 3 shows that the eight women and four men include persons who experience serious difficulty because they are Deaf or hard of hearing, have visual impairment, mobility impairments, or intellectual disabilities. Some of the respondents experience more than a single functional limitation. Table 3 also indicates that most of the respondents had been patients of the Health Center for more than 4 years. All of them had visited the health center in the past 6 months, with the reason for the visit a routine check-up, follow-up for a chronic or acute condition, or recent onset of an acute condition.

**Table 3:** Profile of patients interviewed

Profile characteristic	Number of persons
**Sex**	
Female	8
Male	4
**Functional limitations (ACS disability questions, yes or no to each question)**	
Deaf or serious difficulty hearing	5
Blind or difficulty seeing even with glasses	3
Serious difficulty concentrating, remembering, or making decisions	3
Serious difficulty walking or climbing stairs	7
Difficulty dressing or bathing	4
Difficulty doing errands alone, such as visiting a doctor or shopping	5
**Length of time a patient of the Health Center**	
0–4 years	3
5–9 years	5
> 9 years	4
**How long since most recent visit?**	
0–30 days	4
31–60 days	4
> 60 days	4
**Reason for the most recent visit**	
Follow-up for an acute condition	2
Follow-up for a chronic condition	4
New acute health problem	2
Routine check-up	3
Other	1
**Clearly recalls completing the four accommodation questions on the intake form**	
Yes	2
No	10
**Rating of importance of assistance or accommodation arrangements to sense of receiving good care**	
Very important	12
Somewhat important	0
Not important	0

Two themes emerged from the patient interviews relevant to the role of accommodation information in the EHR: (1) patients did not clearly recall indicating their accommodation need on the registration form and (2) patients expected their health providers to make appropriate accommodation derived from knowledge of the patient and specific requests made prior to the medical visit.

#### Theme 1: patients did not recall indicating an accommodation need on the intake form

Table 3 shows that only 2 of the 12 patients interviewed clearly recalled indicating their accommodation need on the intake form, nor or on any other form. These patients answered the question “No,” did not clearly recall, or answered by talking about their interactions with the Health Center staff to arrange accommodations.

*Question: When you first enrolled in [the Health Center] were you asked whether you would need help during appointments because of limitation in hearing, vision, or movement?*

*Patient 1: I don’t know. They seen my cane and they just helped me. They knew I needed help.*

*Patient 2: It sounds quite possible, but it’s such a long time ago and maybe it’s something that—form I filled out in the initial stages of my participating in their program there that I filled out something like that. But I can’t tell you for sure.*

*Patient 3: Yeah. I had a care worker with me and she helped me through a lot of paper then...*

*Patient 4: I don’t remember being asked in writing or on a form if I need an interpreter. My girlfriend comes with me and she signs during the visit.*

*Patient 5: I would say probably not, no. I don’t remember them asking me anything about my accommodations. And I have a good memory, so, yeah.*

The registration form was not, in their view, the primary means through which they conveyed their accommodation needs to their health providers. The vague recollection of answering the four questions on the registration form suggests that the questions did not stand out from the other background information they were providing. Even the patients who recalled the questions or thought they recalled the questions, did not seem to view them as the Health Center’s primary source of information for arranging visit-related accommodations. Some patients indicated that when they call to schedule an appointment, they remind the scheduler of their accommodation need. However, patients did not view their right to expect accommodation as contingent upon their proactive effort when making an appointment.

#### Theme 2: patients expect their health providers to make appropriate accommodations for their medical visit

Patients expected their health care providers, which included everyone within the medical office, to get to know them, and to implement needed accommodations based upon that knowledge. Several patients gave examples of needed accommodations provided routinely because the Health Center knew them.

*Question: Did you let the Health Center know of any special accommodations before or during your visit?*

*Patient 1: No. They knew I was blind, the ones that knew me. They come out and call my name and, uh—the old ones, who’ve been there, they know me.*

*Patient 7: No, they do that for me [make a longer appointment without having to ask].*

*Patient 5: I guess the only thing—sometimes the nurse will transcribe for me, or they will fill out the boxes for me. That’s like the only accommodation that I really ever say I need. Like, that I need a transcriber.*

Patients who were not completely satisfied with their care experience felt Health Center staff had not invested enough effort to get to know them and to understand their accommodation needs. Other patients indicated that each time they scheduled an appointment they remind the scheduler of their accommodation needs, even if only to confirm what they expect the Health Center to already know.

*Patient 6: My disability is pretty involved and needs diligent care. I am new to California and the clinic did not conduct a medical history. They need to get to know me. I moved back to repair that gap in my healthcare.*

*Patient 8: [She] was saying sometimes when they schedule in advance they say that they can’t accommodate her … So she says normally if [her] needs are mentioned on the phone they will give her a longer appointment. [Patient with assistant on the phone to aid understanding her speech.]*

*Patient 2: Like I kind of suggested, the doctor kind of checked on his computer from his records and it feels to me like he was just sort of pretending to be knowledgeable about my case but it kind of seems like he’s just referring to something he’s just seeing on the computer..*

Patients indicated that knowledge of them is a key source of information about accommodation needs, even if there is information in the formal record. Some patients offered examples of unusual successful accommodations, worked out with health center staff in a collaborative manner. The areas in which patients expressed the greatest frustration were accommodations involving communication. Examples included difficulty using the health center’s web portal, not having materials in Braille, desiring a transcription of the conversation that took place during the visit, and feeling rushed through the appointment (e.g., needing more time for communication).

All the patients indicated that the presence of special assistance or accommodations was very important to their sense of receiving good care. Some elaborated their reasons by speaking about the importance of good communication, whether it involved Sign Language interpretation, written materials, or taking time to really listen. Two patients indicated their understanding that disability accommodation is a right in U.S. law.

*Patient 5: I guess it’s like a—like, if you rate it from like, 1–10, I would say a 7 or an 8 … Well, I have a right to accommodations, so I feel like I should be awarded—I should be given those accommodations if I need them.*

*Patient 12: It’s very important. I’ve been hurting. It’s very important that I have someone to treat me well. Or I wouldn’t even go. I’d find me another doctor.*

*Patient 1: It’s really important. Yeah, because I can’t—I can’t know what room to go in, or get on the exam table by myself. If I’m searching for the table, I might get hurt or fall in the process. Yeah, they’re really nice up there.*

Overall, patients expressed satisfaction with the care they received, and most were satisfied with the accommodations present during their medical visit. However, persons with more complex limitations and needs were more critical of their visit experience. Examples of patients with complex limitations are persons with physical and cognitive limitations, or movement limitations due to combinations of pain, cerebral palsy, or paralysis. These individuals may have required several accommodations, such as assistance getting on and correctly positioned on the exam table, assistance to stand, and a longer appointment slot. This latter accommodation also was important to patients who needed an aide to assist them or interpret their speech to the provider.

## Discussion

This study examined the implementation of four disability-related accommodation need questions in the patient electronic health record system (EHR) of a large primary health care practice. The specific interest was whether and how staff used the accommodation information in the EHR, barriers to its use, and recommendations for the usable inclusion of accommodation needs information. Focus groups gathered data from a mix of Health Center staff. In addition, a selected set of patients were interviewed by telephone. Interviews with patients aimed to learn whether patients recalled answering the four EHR accommodation questions and whether this affected their expectations for disability-related accommodation at a medical visit. The main staff findings suggested that scheduling staff use the EHR-embedded information about disability-related accommodations needs, but clinical staff more often rely on their knowledge of the patient to plan for accommodations during the medical visit. The findings also revealed that structural characteristics of an EHR system affect the usability of the accommodation need information. A key finding from the patient interviews was patients’ general lack of recollection or awareness that they noted accommodation needs as part of initial patient registration. However, patients expected health providers to implement needed disability-related accommodations for their visit.

### Use of EHR accommodation needs information at the medical visit

The inclusion of disability-related accommodation needs in the EHR by the Health Center corresponds to recommendations in the literature that healthcare providers embed information in the EHR to enable advance preparation to treat patients with disabilities [3, 10–12, 14]. These recommendations make the implicit assumption that if the information is present, it will be used. Our findings suggest that use varies strongly by role. The biggest users of the information are front office staff who do not directly deliver health care, but who have tasks to be completed well before the patient arrives. These tasks include recognizing the patient who requires a longer appointment slot and arranging for an ASL interpreter if needed for “effective communication” required by the Americans with Disabilities Act [36]. Accommodation needs come to their attention because responses to the four questions are located in the Patient Management System, the part of the electronic record that serves their roles. To a lesser extent, the medical assistants report using the accommodation need fields to anticipate the allocation of patients to exam rooms with height adjustable exam tables or to identify when additional staff will be needed to assist with a patient.

While disappointing, it may not be surprising that the clinical staff do not routinely review the accommodation need fields in advance of seeing the patient. One reason is that the accommodation information is not located in the part of the patient record the clinical staff routinely use, even when they review a record in advance. No flag or trigger indicating a possible accommodation need appears on the screens these staff typically access either the day before or the day of a patient visit. Without a mechanism drawing attention to a possible need, staff generally will not proactively navigate to a different screen to view the accommodation need information. An accommodation for a regular patient who is known by name is often implemented based upon familiarity with the patient, but even then, it may not be planned well in advance. If an accommodation is required by a new patient, one who is not seen regularly and with whom the staff are not familiar, or one who has acquired a new functional limitation since answering the accommodation questions, it likely will be implemented “on the fly” when the patient arrives for care.

The dependence upon last minute adjustment translates into the experiences reported by patients with disability who describe medical visits in which they were not examined on an examination table or which were limited by a time slot too short to accommodate the extra time required for effective communication or assistance for mobility limitations [2, 6, 37]. Lack of preparation may cause other patients to experience delay, which is associated with dissatisfaction with a care experience [38, 39]. Health Center staff seemed resigned to being behind the projected schedule for patients even while acknowledging this is not desirable. One argument used to advocate for EHRs in general is that they can facilitate a smoother workflow [17, 21, 22]. The Health Center staff discussion indicates embedded accommodation need information also could prevent undesirable delay.

We were surprised that the patients did not clearly recall answering the four questions on the registration form. One reason that the four questions held little significance for patients may be the wording that asked about accommodation need. For example, Health Center staff reported that some patients interpreted the question about accommodation for a mobility limitation as an inquiry about transportation needs. This confusion concurs with prior health setting research examining how patients respond to questions about disability and how they speak about their health conditions and limitations [28, 40, 41]. Answers to, “do you have a disability?” are different from answers to questions asking about limitations in function [41]. With the disability question, patients express confusion over whether their diagnosed condition is a disability or whether their limitation is severe enough to qualify as a disability [40]. If asked in an open-ended fashion about the nature of the disability the answer is sometimes a diagnosis (e.g., Parkinson’s disease) and sometimes a functional statement (e.g., balance problems) [28]. These differences not only highlight the importance of wording, but also the distinction between health condition or diagnosis and functional limitation. To implement an accommodation, knowing the patient’s diagnosis is less useful than knowing the limitation in function. A related thread of research has studied how functional status can be inferred from the medical narrative or the six ACS questions included in EHRs to enable analyses of health equity and the social determinants of health [42–45]. Whether for immediate use to facilitate a medical visit, or for broader understanding of disability and healthcare outcomes, the EHR wording to identify patients’ disability-related functional limitations and needs requires careful consideration.

### Barriers to using EHR embedded accommodation need information

The technical structure of the EHR has a strong impact upon how it is used. A barrier to everyone’s use is the complexity of the software, which requires a significant time investment to become a facile user. Clinical providers must click through a number of screens while interacting with patients, and information that is not flagged or easily accessed may not be reviewed or used [19, 45, 46]. In prior studies physicians report concerns that EHR use increases time spent doing documentation and looking at screens, decreases time in interaction with patients, and negatively affects workflow, a term used to describe the path of people, tasks, and processes that comprise the delivery of care to a patient [18, 22, 46]. Consequently, a structure for entering and reviewing accommodation need that is easy to master and use is necessary to achieve greater documentation. This also will benefit staff members who never meet the patient face-to-face but need to know accommodation needs to schedule appointments or arrange referrals.

One obvious recommendation is that the EPM and EHR be fully integrated. Websites of major EHR software companies suggest that their newest products integrate financial management and patient care information. However, this is only a partial solution if these systems are not structured to collect accommodation need and locate it where staff who need the information can easily access it. Thus far, EHR manufacturers do not offer templates that include accommodation questions.^3^ Healthcare facilities that want to include such questions when they transition to new systems must ask for costly customized solutions. Standardizing accommodation questions in EHR products will facilitate more consistent and widespread use.

Another challenge, evident from the patient interviews, is that patient accommodation needs are not static. The Health Center collected and entered the accommodation need information only once at registration. There was no protocol to regularly update it. The presence and severity of disability can change over time; some limitations diminish, and others expand or become more severe. Analyses of U.S. panel data using the six ACS functional limitation questions found that over a 1 year span, some people consistently report their limitations, but a small percentage report onset or absence of a limitation, such as vision or hearing, that might be expected to change little from year to year [47, 48]. Even without a change in functional status, some accommodations may not be needed at every health visit. For example, a height adjustable exam table may not be necessary when a patient who uses a wheelchair receives care for an ear infection.

### Staff recommendations for structuring EHR accommodation need information

Primary care staff are not expected to be masters of the technical characteristics of EHR systems, but they are users who can offer important guidance regarding the best location, timing, and format of accommodation requirements. The involvement of providers and patients in the identification of EHR capabilities and structure has been recommended by others as a means of increasing EHR buy-in and utilization [26, 49]. A primary recommendation of the Health Center staff was that the accommodation need information be placed where it could be readily noticed by schedulers, nurses, doctors, medical assistants, referral specialists, and others. The standard method in EHRs to remind the user of a patient issue is to put it into an alert. However, systems build in many alerts, creating a risk of one more being ignored [45]. Structural modifications, such as the color coding mentioned by one person, were more appealing to the Health Center staff. The EHR structure also must allow accommodation need to be updated regularly to reflect changes in a patient’s functional status. Staff need to be able to interact easily with the EHR and record changes when they identify a need either during a clinical visit or when the patient makes a request during any point of contact with the medical practice. Finally, while a brief and easy method to enter information is desirable, Health Center staff noted that they sometimes require elaboration of the accommodation need beyond the threshold question. For example, when the need for mobility assistance is checked, should they prepare to use the room with the patient lift or for an additional staff member to assist the patient on and off the examination table?

There is no single point in time when accommodation information is needed because the required actions occur in different time frames. Some accommodations (e.g., scheduling an ASL interpreter) must be arranged well in advance; others can be put in place the day before or day of an appointment; and some require systemic arrangements not tied to a specific patient appointment. For example, when a patient requires use of accessible examination equipment, the time frame for planning varies depending upon how many rooms at the clinic have such equipment. If every room has an accessible exam table, then no advanced planning is required. If there is only one room, then it becomes important to space the appointments of patients needing that room, and to plan for room use the morning of, or 1 day in advance. A survey of California primary care offices associated with Medicaid managed care plans found that few practices have accessible equipment, and those that do, likely have only one height adjustable examination table [8]. Similarly, 1 day’s planning may be required where the assistance of one or more extra staff is needed. Finally, some of the accommodation needs do not require any real time adjustments, but systemic action. An example is producing often-used instructions in alternative communication formats.

Staff interest in knowing how to better serve people with disability corresponds to numerous studies with data from patients and providers that conclude that medical professionals need a greater understanding of disability (as opposed to illness) and more training regarding best practices to deliver primary care to persons with functional limitation [2, 11, 37, 50]. The greater knowledge could lead to greater interest in accommodation planning in advance of a medical visit, with the potential for better health care outcomes.

### Limitations and strengths

This study has limits to generalizability because data were collected from staff and patients of a single health provider organization in a single geographic area. Moreover, the personnel interviewed for this study may not be representative of most primary care office staff because they work for a health center with a focused mission. As noted previously, the FQHC patient census may be disproportionately comprised of persons with low incomes, chronic health problems, and disabilities compared to most primary care practices. Thus, these staff may more often need to utilize accommodations to treat patients. Moreover, the FQHC may attract persons especially dedicated to serving a low income patient population with chronic health problems or disabilities. Finally, not all staff of the targeted clinic sites agreed to participate in a focus group. We do not know what factors differentiate those who volunteered to participate from those who did not. The difference could be convenience and time of day, or it could reflect differences in the level of interest in speaking about patient accommodation information and needs. Similarly, the patients invited to be interviewed were selected for specific characteristics; they cannot be considered representative of all patients or all patients of the Health Center. The response to the interview invitation may reflect an unknown factor affecting willingness to be interviewed about the Health Center. Respondents reported a diversity of functional limitations, with some satisfied and others dissatisfied with their visit logistics, so these factors as bias in selection may be minimal. On the other hand, no respondents were in an acute health crisis, which may have prevented participation of otherwise interested persons.

Researchers also were mindful of the potential for bias in collecting and interpreting data and therefore reflected on this concern throughout the study. In particular, the lead interviewer, who has a background in conducting qualitative descriptive research interviews on diverse topics of concern to people with disabilities, sought feedback from the team in order to ensure that she did not express personal biases that might influence data collection or interpretation. Another limitation is that focus groups and semi-structured interviews do not produce the types of structured data required for statistically generalizing findings to a larger population, whether people or organizations.

A strength of this study is that it offers a unique view of how staff in a busy primary care setting use their electronic records and make real-time adjustments to facilitate the delivery of care to patients with disabilities. While a few studies have examined the workflow of primary care delivery and use of EHRs [45, 51], no prior studies have collected data on the in-situ response to patient accommodation needs. Because to date few large providers of primary care have embedded any accommodation need information into their EHR, this study bridges the gap between the EHR implementation and the disability-accessible health setting literature. The use of an FQHC setting for data collection, while a limitation, also is a strength. With more patients with disabilities, FQHC personnel may have more often observed circumstances where disability accommodation was needed or implemented. Thus, they were able to knowledgeably discuss the interface of EHR use and accommodation implementation. The research setting also demonstrates the importance of leadership that is supportive of staff members’ creative efforts to accommodate patients. Finally, the use of multiple coders who had not participated in the data collection, along with triangulation of information from patients and focus groups, strengthens confidence in the study’s overall conclusions.

For the future, it would be beneficial to have additional studies observe how primary care settings manage their patient flow and implement accommodations for patients who require them. Study of other primary care settings with EHR-embedded accommodation questions would enable development of best practice guidelines. In addition to exploratory and qualitative research, quantitative study of EHR use and patient accommodation would yield insights for structuring EHR systems with patient accommodation information.

## Conclusions

This study offers a unique “on the ground” view of how accommodation need, patient EHR information, and the implementation of accommodations intersect in the delivery of primary care to people with disabilities. The portrait of the daily schedule and patient flow suggest that accommodation information can be useful. However, without an EHR architecture that integrates patient management and patient medical information, use may mostly occur at the point of appointment scheduling. The wording of the accommodation need inquiry is important, as well. Patients must understand what information they are being asked to provide and why. Providers must have sufficient detail about the functional limitation requiring an accommodation to guide planning. Accommodation need may change over time as a patient’s functional abilities change, and so it cannot be collected only once, or only at a single point in a patient’s interaction with a health provider.

This is a timely moment to develop best practices for documenting and implementing patient accommodation need in the EHR as part of meaningful use that supports quality health care delivery.

## Supplementary information

**Additional file 1:** Understanding the Role of Functional Limitation Measures in Electronic Health Records Focus Group Questions – 4.2017

## Data Availability

The qualitative data collected and analyzed from the focus groups and patient interviews are not publicly available. Due to the small setting and small number of informants, this is necessary to maintain confidentiality.

## Abbreviations

EHR: Electronic health record
HITECH: Health Information Technology for Economic and Clinical Health
ASL: American Sign Language
FQHC: Federally Qualified Health Center
IRB: Institutional Review Board
MA: Medical Assistant
EPM: Electronic patient management (record)

## Footnotes

1 The six questions are: (1) Are you deaf, or do you have serious difficulty hearing?, (2) Are you blind, or do you have serious difficulty seeing, even when wearing glasses?, (3) Because of a physical, mental, or emotional condition, do you have serious difficulty concentrating, remembering, or making decisions?, (4) Do you have serious difficulty walking or climbing stairs?, (5) Do you have difficulty dressing or bathing?, and (6) Because of a physical, mental, or emotional condition, do you have difficulty doing errands alone such as visiting a doctor’s office or shopping? Questions are marked yes or no.

2 It is prohibited discrimination under the Americans with Disabilities Act for a medical provider to refuse to treat a patient based upon disability, even if the refusal is due to an inaccessible office .

3 Based upon the authors’ informal inquiries of EHR exhibitors at the 2019 American Public Health Association meeting and communication with Health Center administrative staff.
