# Supplementary material for: Can disability accommodation needs stored in electronic health records help providers prepare for patient visits? A qualitative study
Source: BMC Health Serv Res. 2020 Oct 16;20:958. doi: 10.1186/s12913-020-05808-z (PMC7566113; doi:10.1186/s12913-020-05808-z)
Supplement: Supplementary file 1 — Additional file 1: Understanding the Role of Functional Limitation Measures in Electronic Health Records Focus Group Questions – 4.2017 [file 12913_2020_5808_MOESM1_ESM.docx]

**Understanding the Role of Functional Limitation Measures in Electronic Health Records** Focus Group Questions – 4.2017

Introduction

--Introduce that the 4 questions are asked and in which data base the answers are stored (in the EPM, under “Patient Information” – there are two fields: “Assistance During Appointment” and “Additional Appointment Assistance”)

--Explain why there is interest in understanding how accommodation information is collected and used

--We are interested in learning what you use, what is useful, what you recommend

Q: The first questions are about if you have a patient in who has an accommodation need for a mobility or other limitation

1. Do you usually know that a patient has an accommodation need ahead of the appointment?
   1. If this is a new patient, how do you know you have a patient with an accommodation need?
   2. If this is a continuing patient, how do you know this patient has an accommodation need?
2. If the patient tells you he/she needs an accommodation, where do you document that?
3. At what point do you need to know about a patient’s accommodation need?
   1. Does it matter how a new patient has registered? Same day as the appointment, on the phone, …..?
4. If you know about the accommodation need ahead of the appointment, do you do any planning for it in advance?
   1. Who is responsible for doing the planning, making the arrangements?
   2. How does the patient’s type of accommodation need affect planning?
5. Where is the usual place/database you use to get the information you need for planning patient visits or services?
   1. Do you ever look at the accommodation fields in the EPM?
6. How much importance is usually placed on accommodation needs in comparison to other factors when scheduling?
   1. If you notice the notation of mobility limitation or the need extra time for assistance, does that affect scheduling?
   2. Does it affect planning for the day
   3. What is the procedure if the patient record indicates the need for sign language interpretation?
   4. Is there any training regarding what actions the information about accommodations should trigger?

Q: What are the general challenges and issues, including logistics and scheduling, that are part of preparation for patients (those with disabilities and accommodation needs AND those without accommodation needs)?

1. When a patient arrives who requires additional time or a height adjustable exam table, and you did not know ahead of time, what factors affect making those accommodations possible at point of service?
2. How often does that happen?
3. If you don’t have information about the patient’s accommodation need ahead of time, how does that affect what you can do during a medical appointment?
4. What kinds of accommodations do you most often have to deal with on the fly?
5. If a desirable accommodation is not present (e.g., it’s not possible to extend the exam time), how do you usually handle the situation?
6. How do a patient’s accommodation needs affect the work flow of the clinic?

Q: What is your ideal scenario for the information you need about a patient to deliver quality care to patients who have mobility, vision, or other disabilities that can require more time or accessible equipment in order to treat them?

1. Where/in what data tool would you like this information?
2. What types of information would you like to have and at what point in the process would you like to have the information?
3. What information would be useful at the point of making an appointment? Where in [EHR product] would you like it to be? Is there somewhere else it should be, as well, or instead of?
4. What information would be useful when the patient arrives for the appointment (in what data tool)?
5. What information would be useful during the exam or treatment (in what data tool)?
6. If you perceive a patient need for accommodation, what would be process to document it to assist planning for future visits?
7. Where would you document a new accommodation need (whether from patient request or provider observation) so that information would be available for a future appointment?
8. Where should that be documented? (e.g., where it should be documented is different than where it would be documented, should it be documented in more than one place?)

Q: In your experience, what works well with respect to getting the information you need to treat a patient with accommodation needs?

1. In your experience, what are the most important (or difficult) barriers to conducting a thorough medical exam on a patient with disabilities?
